# Supplementary material for: Inclusion of the Woodchuck Hepatitis Virus Posttranscriptional Regulatory Element Enhances AAV2-Driven Transduction of Mouse and Human Retina
Source: Mol Ther Nucleic Acids. 2016 Dec 31;6:198–208. doi: 10.1016/j.omtn.2016.12.006 (PMC5363497; doi:10.1016/j.omtn.2016.12.006)
Supplement: Document S1. Tables S1–S3 [file mmc1.pdf]

OMTN, Volume *6*

Supplemental Information

Inclusion of the Woodchuck Hepatitis Virus Posttranscriptional Regulatory Element Enhances AAV2-Driven Transduction of Mouse and Human Retina

Maria I. Patrício, Alun R. Barnard, Harry O. Orlans, Michelle E. McClements, and Robert E. MacLaren

Supplemental Methods

Table S1 – Total protein loading amounts and antibodies dilutions' used for Western blot analysis for AAV2-GFP±WPRE treated samples.

**Antibody/Sample type AAV2-GFP±WPRE**	AAV-transduced 293 cells	AAV-injected eye cups	AAV-injected retinas
Total amount of protein loaded	5 µg	1 µg	4 µg
Dilution of anti-GFP (ab32146, Abcam, Cambridge, UK)	1:20,000	1:10,000	1:10,000
Dilution of anti-β-Actin (AM4302, Thermo-Fisher Scientific, Loughborough, UK)	1:20,000	1:10,000	1:10,000
Dilution of Donkey anti-mouse HRP (ab98799, Abcam, Cambridge, UK)	1:10,000	1:10,000	1:10,000
Dilution of Donkey anti-rabbit HRP (ab98503, Abcam, Cambridge, UK)	1:10,000	1:10,000	1:10,000

Table S2 - Total protein loading amounts and antibodies dilutions' used for Western blot analysis for AAV2-REP1±WPRE treated samples.

**Antibody/Sample type AAV2-REP1±WPRE**	AAV-transduced 293 cells	AAV-injected eye cups	AAV-injected retinas
Total amount of protein loaded	10 µg	10 µg	25 µg
Dilution of anti-human REP1 (MABN52, Millipore, Watford, UK)	1:2,500	1:1,000	1:1,000
Dilution of anti-β-Actin (AM4302, Thermo-Fisher Scientific, Loughborough, UK)	1:50,000	1:50,000	1:50,000
Dilution of Donkey anti-mouse HRP (ab98799, Abcam, Cambridge, UK)	1:10,000	1:10,000	1:10,000
Dilution of Donkey anti-rabbit HRP (ab98503, Abcam, Cambridge, UK)	1:10,000	1:10,000	1:10,000

Table S3 – List of antibodies used in immunohistochemistry analysis.

**Antibodies**	Dilution
Anti-cone arrestin (AB15282, Millipore, Watford, UK)	1:500
Anti-GFAP (ab7779, Abcam, Cambridge, UK)	1:500
Anti-CHM (HPA003231, Sigma, Gillingham, UK)	1:1,000
Donkey anti-rabbit Alexa Fluor 568 (A10042, Thermo-Fisher Scientific, Loughborough, UK)	1:500
