# Supplementary material for: Inclusion of the Woodchuck Hepatitis Virus Posttranscriptional Regulatory Element Enhances AAV2-Driven Transduction of Mouse and Human Retina
Source: Mol Ther Nucleic Acids. 2016 Dec 31;6:198–208. doi: 10.1016/j.omtn.2016.12.006 (PMC5363497; doi:10.1016/j.omtn.2016.12.006)
Supplement: Document S2. Article plus Supplemental Information [file mmc2.pdf]

Molecular Therapy
Nucleic Acids
Original Article

Inclusion of the Woodchuck Hepatitis Virus Posttranscriptional Regulatory Element Enhances AAV2-Driven Transduction of Mouse and Human Retina

Maria I. Patrício,[1,2,3] Alun R. Barnard,[1,2,3] Harry O. Orlans,[1,2,3,4] Michelle E. McClements,[1,2] and Robert E. MacLaren[1,2,3,4]

[1]Nuffield Laboratory of Ophthalmology, Nuffield Department of Clinical Neurosciences, University of Oxford, Oxford OX3 9DU, UK; [2]NIHR Oxford Biomedical Research Centre, University of Oxford, Oxford OX3 9DU, UK; [3]Oxford Eye Hospital, Oxford University Hospitals NHS Foundation Trust, Oxford OX3 9DU, UK; [4]Moorfields Eye Hospital, NHS Foundation Trust, London EC1V 2PD, UK

The woodchuck hepatitis virus posttranscriptional regulatory element (WPRE) has been included in the transgene cassette of adeno-associated virus (AAV) in several gene therapy clinical trials, including those for inherited retinal diseases. However, the extent to which WPRE increases transgene expression in the retina is still unclear. To address this question, AAV2 vectors containing a reporter gene with and without WPRE were initially compared in vitro and subsequently in vivo by subretinal delivery in mice. In both instances, the presence of WPRE led to significantly higher levels of transgene expression as measured by fundus fluorescence, western blot, and immunohistochemistry. The two vectors were further compared in human retinal explants derived from patients undergoing clinically indicated retinectomy, where again the presence of WPRE resulted in an enhancement of reporter gene expression. Finally, an analogous approach using a transgene currently employed in a clinical trial for choroideremia delivered similar results both in vitro and in vivo, confirming that the WPRE effect is transgene independent. Our data fully support the inclusion of WPRE in ongoing and future AAV retinal gene therapy trials, where it may allow a therapeutic effect to be achieved at an overall lower dose of vector.

INTRODUCTION

Inherited retinal dystrophies (IRDs) are monogenic diseases caused by mutations in genes crucial for the function of retinal cells, most commonly photoreceptors and/or the retinal pigment epithelium (RPE). IRDs can result in sight loss and affect ~1 in 4,000 people worldwide.[1] The identification of many IRD-causing genes has paved the way for the development of treatment strategies, including gene-therapy-based approaches involving gene replacement.

The fact that the eye is a relatively immune-privileged site makes retinal gene therapy an attractive prospect. The target cells of the retina are differentiated and non-dividing, making them ideal candidates for non-integrating viral systems such as adeno-associated virus (AAV), which may achieve continued gene expression. Recombinant AAV strategies have been the prime choice for retinal gene therapy due to their favorable toxicity profile and benign immune response.[2,3] Since the finding that AAV can transduce photoreceptor cells following subretinal injection,[4] several groups have tested both wild-type and mutant AAV capsids of various serotypes to maximize simultaneous transduction of RPE and photoreceptors.[5–8] The demonstration of safety and efficacy of serotype 2 in a canine model of Leber congenital amaurosis (LCA)[9] justified its used in the first AAV gene therapy trials in the human eye.[10–12] Other diseases followed, and currently, there are several ongoing clinical trials delivering wild-type AAV2 by subretinal injection.[13–16]

The results of a phase 1/2 trial of gene therapy for LCA showed improved retinal sensitivity; however, cases of inflammation and/or immune responses were reported,[17,18] highlighting the issue of AAV immunogenicity. Improving vector efficiency through inclusion of a posttranscriptional regulatory element (PRE) within the transgene cassette may help overcome this problem by allowing a lower dose of AAV to be delivered to achieve any given treatment effect. The woodchuck hepatitis virus (WHV) posttranscriptional regulatory element (WPRE) has been shown to enhance AAV transgene expression,[19–24] and it is included in Glybera, the first gene therapy drug approved in the European Union.[25] Its potent activity over other regulatory elements is suggested to be due to the unique fact that it increases mRNA levels by enhanced transcript termination.[26,27]

A WPRE-containing AAV2 transgene was found to successfully transduce both RPE and photoreceptors in human retinal explants

Received 18 November 2016; accepted 13 December 2016;
http://dx.doi.org/10.1016/j.omtn.2016.12.006.

Correspondence: Robert E. MacLaren, Nuffield Laboratory of Ophthalmology, University of Oxford, John Radcliffe Hospital, Headley Way, Oxford OX3 9DU, UK.
E-mail: enquiries@eye.ox.ac.uk

This is an open access article under the CC BY license (http://creativecommons.org/licenses/by/4.0/).

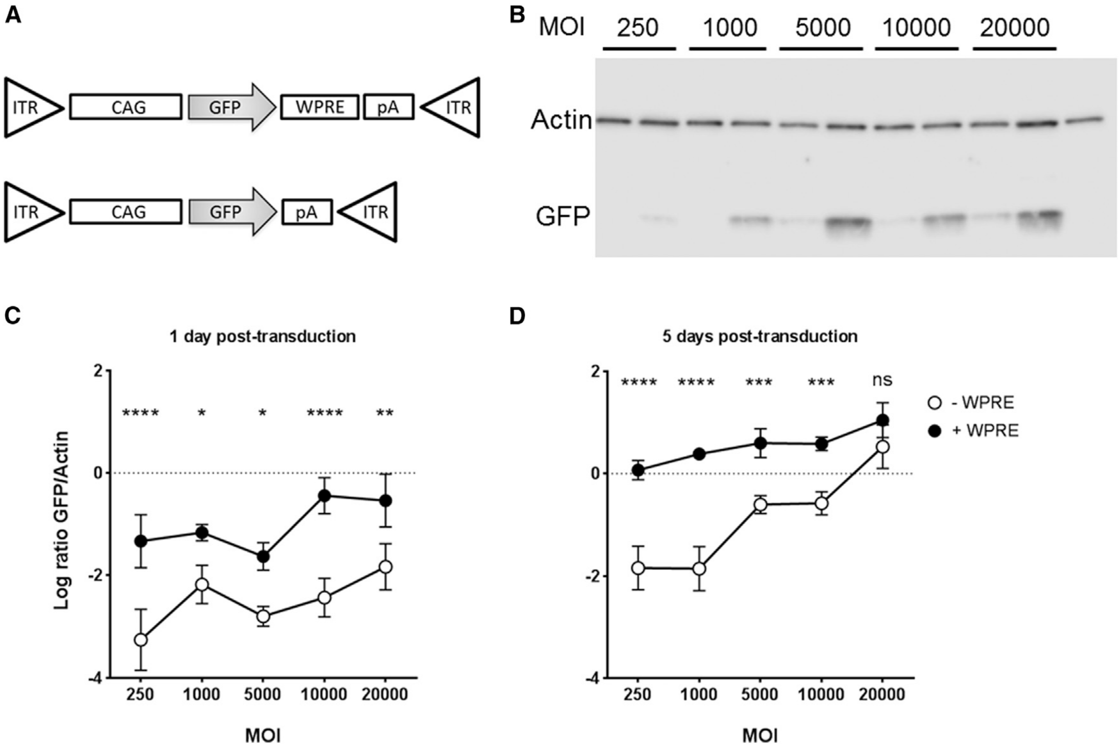

Figure 1. Plasmid Design and Transgene Expression following AAV2 Transduction In Vitro

(A) Design of the pAAV2-GFP and pAAV2-GFP-WPRE vectors. The expression cassette contains the following DNA sequences: ITR, AAV2 inverted terminal repeats; CAG, hybrid of the human cytomegalovirus upstream enhancer with the chicken β-actin promoter; CHM or GFP, DNA coding sequences for REP1 and GFP proteins, respectively; WPRE, woodchuck hepatitis virus posttranscriptional regulatory element; pA, bovine growth hormone polyadenylation signal. (B) Representative western blot data from 293 cells transduced with pAAV2-GFP (–) and pAAV2-GFP-WPRE (+) at a range of MOI and harvested at 5 days post-transduction. Untransduced cells were used as a negative control for GFP expression. Actin was used as loading control. (C and D) western blot quantification data from 293 cell transduction with pAAV2-GFP ± WPRE. Cells were harvested at 1 (C) and 5 (D) days post-transduction. GFP expression levels were detected by WB and normalized to actin as a loading control. Symbols are log-base-10-transformed mean values of three replicates ± SEM. The effect of the presence of WPRE for each MOI was analyzed by two-way ANOVA, with Bonferroni's multiple comparisons test. *$p < 0.05$; **$p < 0.01$; ***$p < 0.001$; ****$p < 0.0001$; ns, not significant.

ex vivo and following subretinal injections in mice.[28] Although the WPRE is widely used in a variety of gene therapy trials, including a phase 1/2 clinical trial for choroideremia,[13] it is still not clear the degree by which it increases transgene expression in retinal cells. The purpose of this study was to investigate the effect of the WPRE on AAV2-delivered transgene expression in vitro, the murine retina in vivo, and the human retina ex vivo by comparing constructs with and without WPRE side by side. We generated AAV2 vectors containing the GFP reporter gene for in vivo monitoring and quantification of expression levels in the retina over time. Previous studies have implemented standardized fundus autofluorescence (AF) imaging in mice and established a method for quantification.[29,30] This provides a unique non-invasive way to assess levels of fluorescence in the retina in vivo. In this study, in vivo comparisons were performed at two different doses of AAV, and we studied how the WPRE influences the expression pattern within the retinal layers. The same experimental approach was then performed using *CHM* cDNA—the gene responsible for choroideremia, formerly known as *REP1* (Rab escort protein 1)—as representative of a clinical scenario. We also investigated efficacy of the WPRE in the human retina using retinal tissue extracted from patients undergoing clinically indicated retinectomy. Together, these data provide evidence that the inclusion of the WPRE within a given AAV2 transgene cassette significantly enhances expression of the target gene within the retina following transduction.

RESULTS

Generation of AAV Vectors

The AAV2 transgene plasmid in use in an ongoing choroideremia gene therapy phase 1/2 clinical trial (NCT01461213) was previously designed to express REP1 in the most efficient way: it contains a ubiquitous CAG promoter, Kozak consensus sequence, WPRE sequence, and a bovine growth hormone polyadenylation signal (pAAV2-CAG-REP1-WPRE-pA).[13,31] To investigate the effects of the WPRE in cell transduction in vitro and in vivo, the original plasmid was modified to express GFP by excision of human *CHM* cDNA, with and without WPRE: pAAV2-CAG-GFP-WPRE-pA and pAAV2-CAG-GFP-pA (Figure 1A). Viral transgenes were packaged into AAV serotype 2 capsids and purified according to established protocols.[32] The number of genome copies (gc) per milliliter was determined by qPCR.

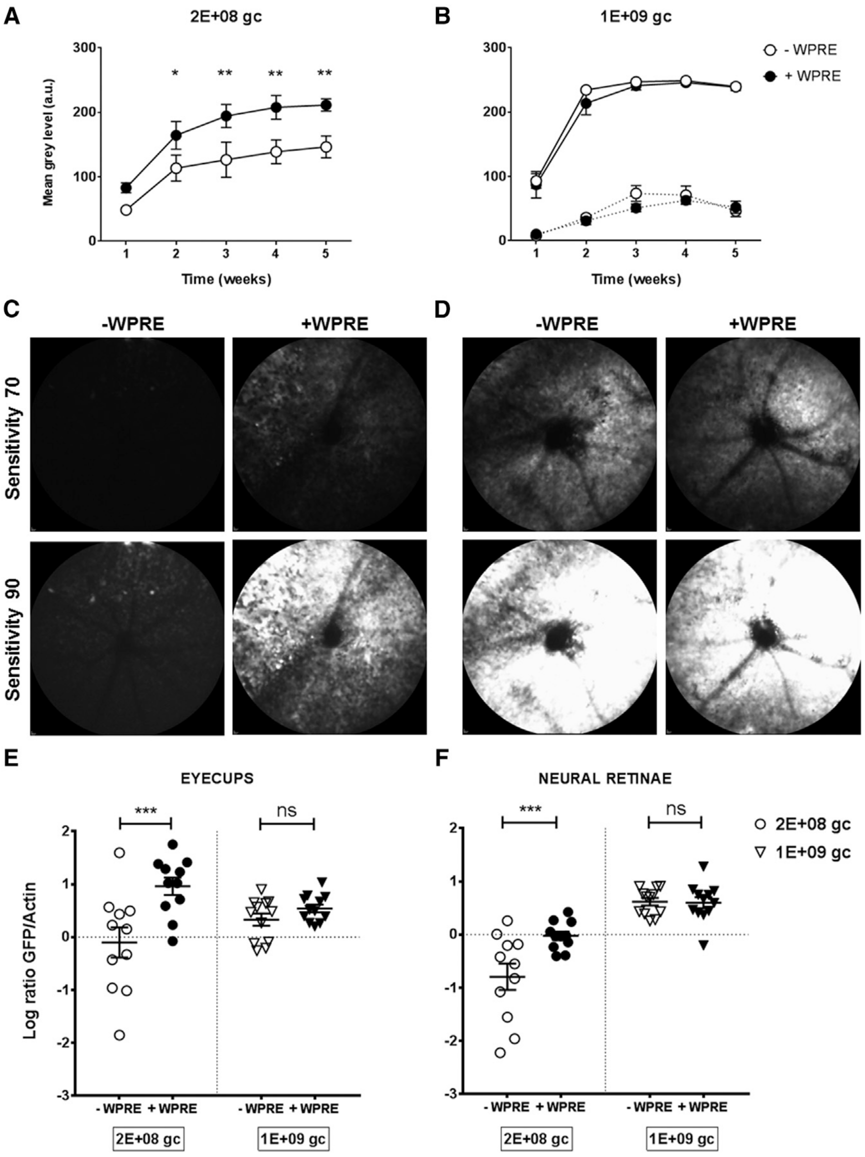

Figure 2. Quantification of GFP Expression Levels In Vivo following Subretinal Injection of AAV2-GFP ± WPRE in C57BL/6J Mice and WB Analysis at 5 Weeks

(A–D) cSLO AF images from mice fundus were processed, and data were plotted over time for 2E+08 gc (A) and 1E+09 gc (B) doses of AAV2-GFP ± WPRE. Dashed lines in (B) refer to images taken at a lower detector sensitivity than the continuous lines in (A) and (B). Symbols are mean values from six animals ± SEM. The effect of the WPRE at each time point was analyzed by repeated-measures two-way ANOVA with Bonferroni's multiple comparisons test. Representative cSLO images are shown for one 2E+08-injected (C) and one 1E+09-injected (D) animal at 4 weeks post-injection at a detector sensitivity of 70 and 90. (E and F) Two cohorts of C57BL/6J eyes were injected bilaterally with AAV2-GFP ± WPRE at 2E+08 gc and 1E+09 gc. Eyes were harvested for detection of GFP levels in eyecups (E) and neural retinae (F) (n = 11); actin was used as a loading control for data normalization. Symbols are log-base-10-transformed values ± SEM. The effect of the WPRE on transgene expression was analyzed by two-way ANOVA with Bonferroni's multiple comparisons test; *p < 0.05; **p < 0.01; ***p < 0.001; ns, not significant.

Both viral preparations were tested in vitro prior to in vivo studies to confirm AAV infectivity and transgene expression. 293 cells were transduced with both vectors at a range of MOIs (250, 1,000, 5,000, 10,000, and 20,000), and transgene expression was detected by western blot (WB) at 1 and 5 days post-transduction (Figures 1B–1D). A two-way ANOVA with MOI and WPRE as factors found that both were highly significant at day 1 post-transduction ($p = 0.0003$ for MOI, $p < 0.0001$ for WPRE) and day 5 post-transduction ($p < 0.0001$ for both MOI and WPRE). Bonferroni's multiple comparison tests for the effect of the WPRE at a given MOI showed a significant pairwise difference in all analyzed at day 1 (250, $p < 0.0001$; 1,000, $p = 0.03$; 5,000, $p = 0.01$; 10,000, $p < 0.0001$; and 20000, $p = 0.0043$). For day 5 post-transduction, all MOI except for 20000 showed a significant increase in GFP expression in the presence of the WPRE (250 and 1,000, $p < 0.0001$; 5,000, $p = 0.0004$; 10,000, $p = 0.0005$; and 20,000, not significant [ns]). Overall, these data show that the presence of the WPRE contributes to a more robust expression of GFP over a wider range of MOIs in vitro.

In Vivo Transgene Expression Levels following AAV Delivery by Subretinal Injection

The influence of the WPRE was then assessed in vivo, a scenario with relevance for clinical trials. We were specifically interested in the effect of the WPRE in photoreceptor transgene expression. Previous work has shown high levels of GFP expression following subretinal injection of an AAV2-GFP vector at 1E+09 gc after 3 weeks[30] as assessed by in vivo confocal scanning laser ophthalmoscopy (cSLO) imaging. In anticipation of high levels of GFP expression in the retina, we also injected a lower dose for each vector. Two cohorts of animals were bilaterally injected with AAV2-GFP-WPRE and AAV2-GFP: one cohort at 2E+08 gc and one cohort at a higher dose of 1E+09 gc. GFP fluorescence was recorded by cSLO weekly from week 1 post-injection up to 5 weeks, analyzed, and plotted as mean gray level (Figures 2A–2D). The first observation was that following a single superior subretinal injection, GFP expression could be detected all over the retina and was not limited to the injection site. Second, different sensitivities of the detector were tested to find the best dynamic range for GFP expression measurement and comparison between doses. Results from a

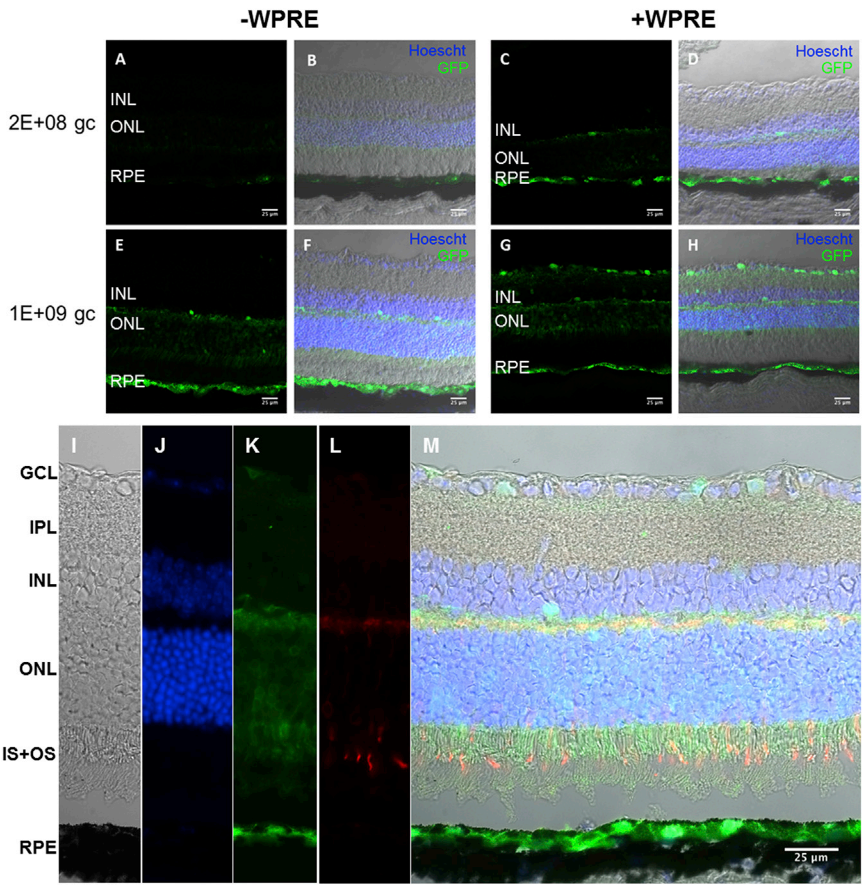

Figure 3. Effect of WPRE in Expression Patterns of GFP in Retinal Layers following Subretinal Injection of AAV2-GFP ± WPRE in C57BL/6J Mice

(A–H) Confocal stacks were prepared from histological sections from eyes injected with AAV2-GFP ± WPRE at 2E+08 gc (A–D) and 1E+09 gc (E–H). GFP signal (green) and nuclear labeling with Hoechst (blue) were overlaid with the differential interference contrast (DIC) image to demonstrate the retinal layers. Scale bar, 25 μm. (I–M) Representative confocal slices were prepared from histological sections of an AAV2-GFP-WPRE-injected eye (1E+09 gc). GFP signal (K) and nuclear labeling (J) were overlaid with immunostaining using cone arrestin (L) and DIC (I) to demonstrate that GFP is expressed mainly in the RPE and rod outer segments. Scale bars, 25 μm. Detached areas are an artifact of the processing steps. GCL, ganglion cell layer; IPL, inner plexiform layer; INL, inner nuclear layer; ONL, outer nuclear layer; IS+OS, inner and outer segments of photoreceptors; RPE, retinal pigment epithelium.

repeated-measures two-way ANOVA analysis with time and WPRE as factors showed that for the 1E+09 gc-injected cohort, only time is a significant factor ($p < 0.0001$) at both sensitivity 60 (dashed lines in Figure 2B) and sensitivity 90 (continuous lines in Figure 2B). At this dose, Bonferroni's multiple comparisons tests did not indicate any significant difference in pairwise comparisons at any individual time point. For the lower dose, analysis of images taken at the same higher sensitivity settings showed that both time ($p < 0.0001$) and WPRE ($p = 0.00492$) were significant factors (Figure 2A). Moreover, from week 2 onward, the +WPRE-injected eyes showed significantly higher mean gray level as confirmed by Bonferroni's multiple comparisons tests. At a dose of AAV2 that we know to be highly efficient for outer retina transduction (1E+09 gc), the presence of the WPRE did not significantly influence the level of GFP transduction, suggesting a ceiling or saturation effect. At a five-times-lower dose (2E+08 gc), however, the effects of the WPRE were to significantly enhance the observed GFP expression, such that by 5 weeks, the fluorescent signal was similar to the level obtained with the higher dose. Our results show for the first time that the presence of the WPRE significantly increases the expression of GFP in vivo following AAV2 subretinal delivery.

After 5 weeks, eyes were processed for either WB or histological analysis. The investigators were blinded with regard to which of the viruses contained WPRE until after all analyses were complete. For WB analysis, eyecups and neural retinae were processed and probed separately for GFP (Figures 2E and 2F). A two-way ANOVA with WPRE and dose as factors revealed that both were significant in regard to the retinae ($p = 0.0103$ for WPRE, $p < 0.0001$ for dose); for eyecups, only the WPRE influence was significant ($p = 0.0007$). Bonferroni's multiple comparison test for the effect of the WPRE at a given dose in both tissues corroborated the findings of cSLO image quantification regarding the 1E+09 gc dose cohort, and no significant difference was found in either eyecups ($p = 0.7653$) or retinae ($p > 0.9999$). By contrast, for the lower-dose cohort, the presence of the WPRE significantly increased GFP expression in both eyecups ($p = 0.0002$) and retinae ($p = 0.0009$).

Histological analysis of injected eyes showed the RPE to be the main target of the AAV2 vectors following subretinal delivery (Figure 3). Images were acquired using identical settings and GFP expression was detected with both doses and constructs used (Figures 3A–3H). There was a visible increase in GFP expression in the outer nuclear layer (ONL) when 1E+09 gc of AAV was delivered, compared to the lower dose (Figure 3E). The presence of the WPRE in the high-dose cohort drove GFP expression in the inner retinal layers (Figure 3G), which was not seen without the WPRE (Figure 3E). Sections of the higher-dose-injected retinae were then analyzed in more detail (Figures 3I–3M). Within the transduced regions, and in addition to the extensive labeling of the RPE, we observed extensive labeling of photoreceptor cells in the ONL with GFP (Figure 3K), thus confirming the suitability of this particular AAV2 expression cassette for transgene expression in both layers. Immunostaining of GFP-injected sections with cone arrestin showed

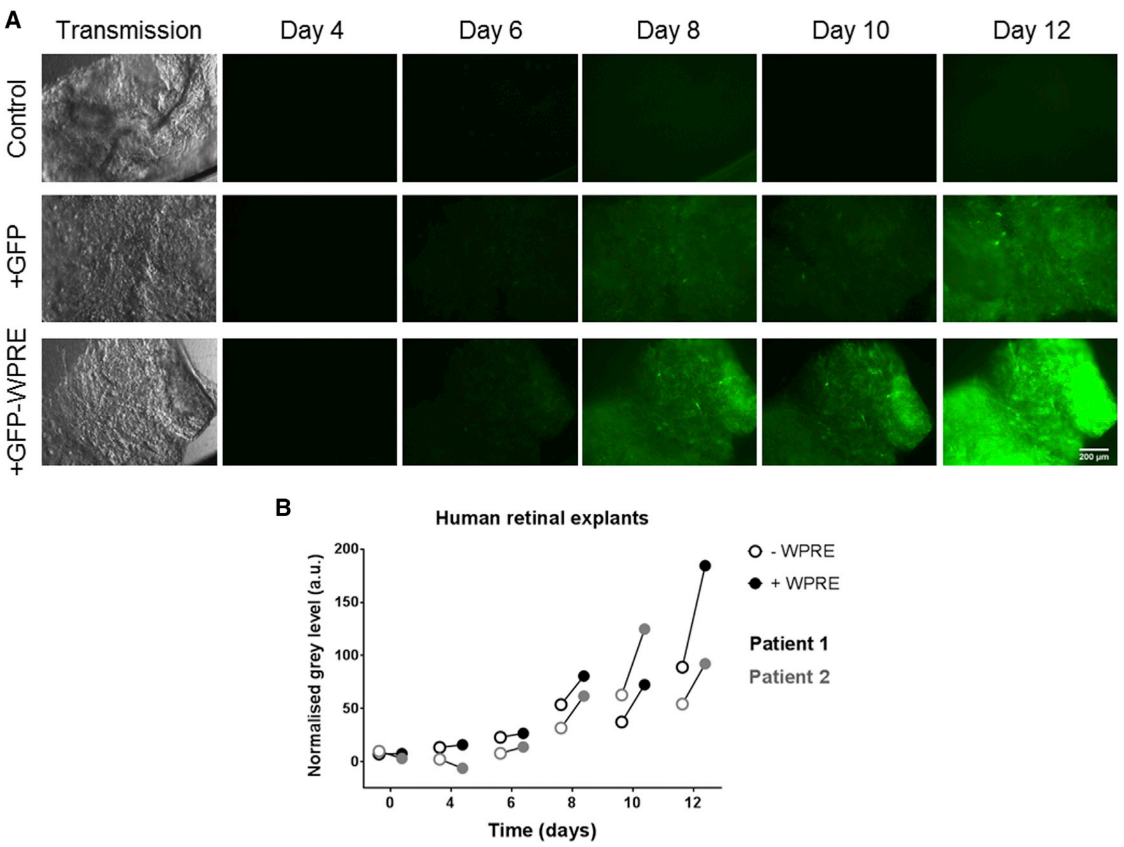

Figure 4. GFP Fluorescence following Ex Vivo Administration of AAV2-GFP ± WPRE in Human Retinal Tissue

(A) Transmission and fluorescence images were acquired on alternate days post-transduction with AAV2-GFP and AAV2-GFP-WPRE under standardized conditions. A non-transduced sample was imaged in parallel as a control. Normalized gray levels were calculated by subtracting the mean gray level for the control sample fluorescent image from that obtained from the equivalent image of transduced retina. (B) Normalized gray levels of two human retinal explants (patient 1, black circles; patient 2, gray circles) transduced with AAV2-GFP ± WPRE were plotted over time showing a trend for increased GFP expression in the presence of the WPRE. Scale bar, 200 μm.

evidence that the rod photoreceptors were the main target photoreceptor cells, although co-localization was detected in some cone cells (Figure 3L).

The WPRE Effect in the Transduction of Human Retinal Explants Ex Vivo

In order to assess whether the WPRE would also benefit transgene expression following AAV transduction in human tissue, samples of human retina were obtained from two patients undergoing clinically indicated retinectomy. The human retinal tissue fragments were maintained in an ex vivo culture system, as described previously.[28,33] The tissue samples were transduced in a masked fashion with either AAV2-GFP or AAV2-GFP-WPRE, with a further sample maintained as an untransduced control, and GFP fluorescence was monitored for 12 days. Representative images are shown in Figure 4. This extracted tissue was macroscopically atrophic but appeared to survive well in culture. GFP expression, as quantified using the normalized mean gray level, was consistently higher in fragments transduced by the vector containing WPRE (Figures 4A and 4B), confirming that WPRE also has a positive effect on transgene expression in human retinal tissue.

CHM Transgene Expression as an Example of Clinical Applicability and Relevance

The use of GFP for transgene expression studies in the retina is very useful, as it allows in vivo monitoring of protein levels over time and their quantification; however, GFP-tagged expression cassettes are not appropriate for clinical trial studies. Therefore, we decided to investigate the effect of the WPRE in a more clinically relevant gene expression cassette with valuable outcome with regard to retinal cell transduction. We utilized the vector currently in use in a gene therapy trial for choroideremia[13,31] plus a modified version prepared by excision of the WPRE sequence. AAV2 vectors were prepared for both versions, titered, and validated in vitro as described previously. 293 cells were transduced with both vectors at a range of MOIs (250, 1,000, 5,000, 10,000, and 20,000), and REP1 expression was detected by WB at 1 and 5 days post-transduction (Figures 5A–5C). 293 cells express REP1 endogenously (gray bar in Figures 5B and 5C), but

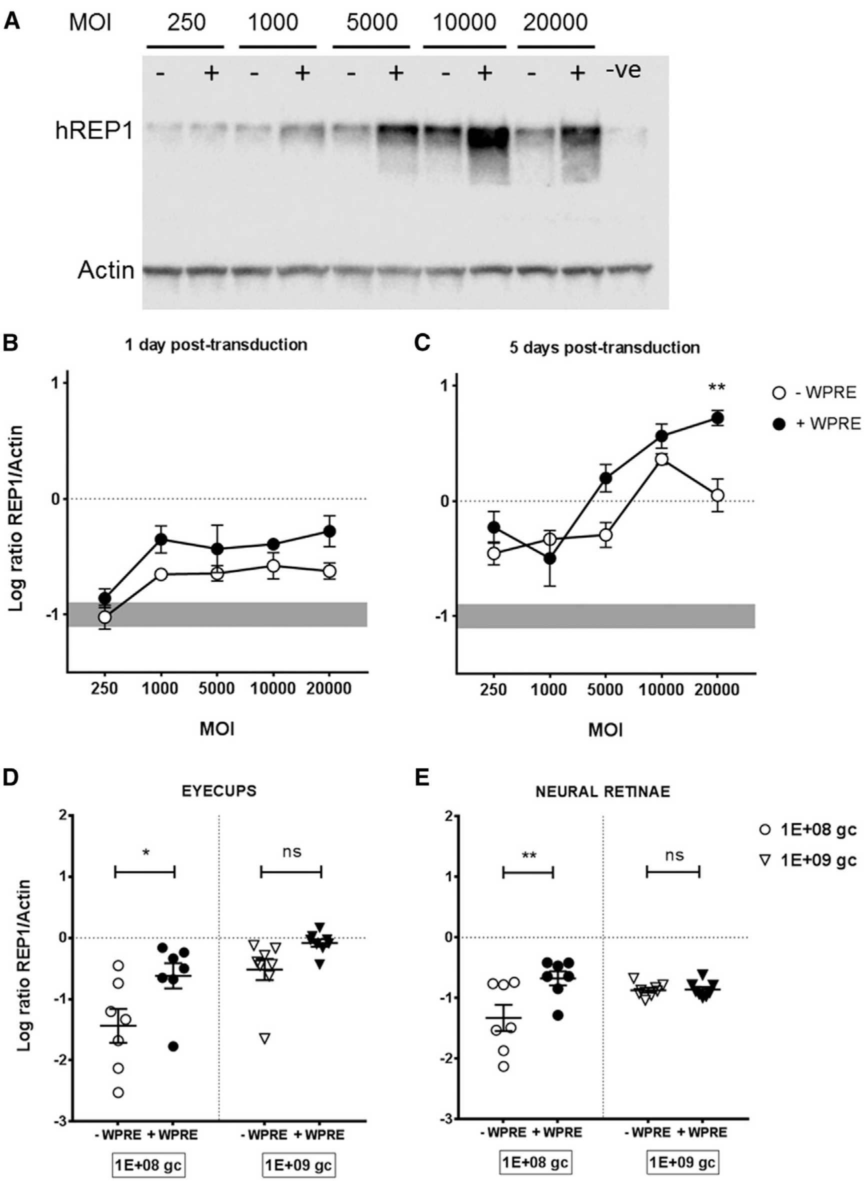

Figure 5. *CHM* Transgene Expression In Vitro and WB Analysis in Eyecups and Retinae 5 Weeks following Subretinal Injection of AAV2-REP1 ± WPRE in C57BL/6J Mice

(A) Representative western blot data from 293 cells transduced with AAV2-REP1 (−) and AAV2-REP1-WPRE (+) at a range of MOI and harvested at 5 days post-transduction. Untransduced cells were used as a negative control for human REP1 expression. Actin was used as a loading control. (B and C) western blot quantification data from 293 cell transduction with AAV2-REP1 ± WPRE. Cells were harvested at 1 (B) and 5 (C) days post-transduction. Human REP1 expression levels were detected by WB and normalized to actin as loading control. The gray bar in (B) and (C) refers to the endogenous levels of REP1 at the time of harvesting. Symbols are log-base-10-transformed mean values of three replicates ± SEM. The effect of the presence of WPRE for each MOI was analyzed by two-way ANOVA with Bonferroni multiple comparisons test. (D and E) Two cohorts of C57BL/6J eyes were injected bilaterally with AAV2-REP1 ± WPRE at 1E+08 gc and 1E+09 gc. Eyes were harvested for detection of human REP1 levels in eyecups and neural retinae (n = 7). Actin was used as a loading control for data normalization. Symbols are log-base-10-transformed values ± SEM. The effect of the WPRE on transgene expression was analyzed by two-way ANOVA with Bonferroni's multiple comparisons test. *$p < 0.05$; **$p < 0.01$; ns, not significant.

transgene REP1 expression levels were detected above baseline. A two-way ANOVA with MOI and WPRE as factors found that both were significant at day 1 post-transduction (p = 0.001 for MOI, p = 0.0019 for WPRE) and day 5 post-transduction (p < 0.0001 for MOI, p = 0.0016 for WPRE). Bonferroni's multiple comparison tests for the effect of the WPRE at a given MOI did not show any significant pairwise difference in all analyzed at day 1. At day 5 post-transduction, REP1 levels were increased above baseline for all tested MOIs; however, only MOI 20,000 showed a significant increase in REP1 expression in the presence of the WPRE following pairwise analysis (p = 0.005). These data reveal increased REP1 expression in the presence of the WPRE in vitro, corroborating the previous findings with GFP vectors.

Following the successful in vitro testing, two separate groups of animals were then injected with AAV2-REP1 ± WPRE at two different doses. In this experiment, the high dose was 1E+09 gc (as for the GFP study), but a 10-fold-lower dose was chosen (1E+08 gc) to better mimic the clinical setting in which a dose escalation is on trial (NCT01461213).[13] Eyes were processed either for WB or histological analysis 5 weeks post-injection, and the investigators were once again blinded with regard to which of the vectors contained WPRE until all analyses were complete. Eyecups and neural retinae were processed and probed separately for REP1 (Figures 5D and 5E). Overall the ratio of transgene to endogenous control was lower than that detected for GFP, even for the higher-dose cohort. A two-way ANOVA with WPRE and dose as factors revealed that both significantly increased REP1 in the eyecups (p = 0.0026 for WPRE, p = 0.0007 for dose); in the retinae, only the WPRE influence on REP1 expression was significant (p = 0.0097). Bonferroni's multiple comparison test was performed to determine the effect of the WPRE at a given dose in both tissue samples, and the results corroborated the findings from the GFP study, as no significant effect was found in either eyecups (p = 0.2074) or retinae (p > 0.9999) at the higher dose of AAV. For the lower-dose cohort, the presence of the WPRE significantly increased REP1 expression in both eyecups (p = 0.0124) and retinae (p = 0.0017).

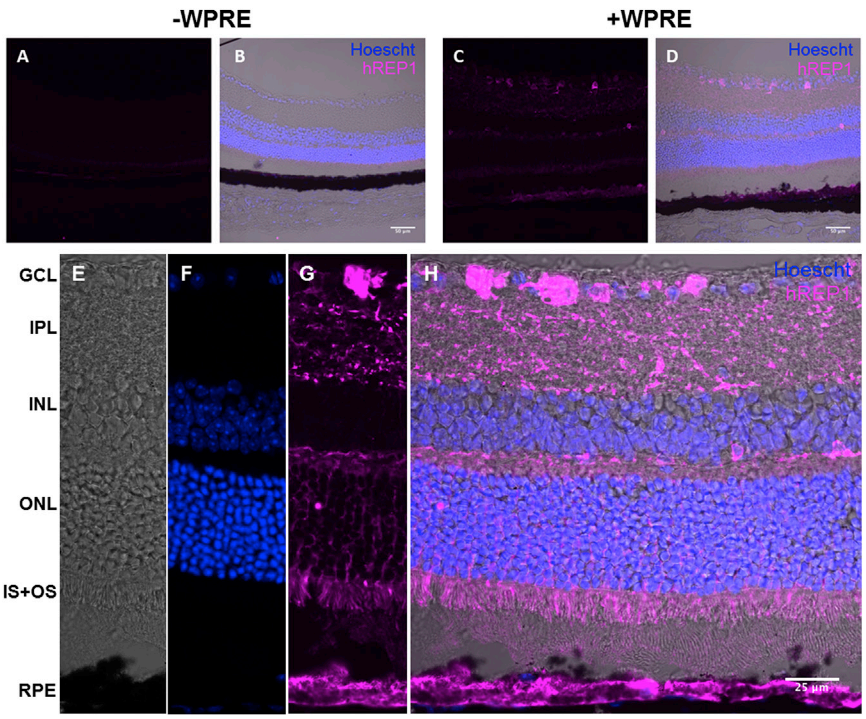

Figure 6. Effect of WPRE in Expression Patterns of Human REP1 in Retinal Layers 5 Weeks following Subretinal Injection of AAV2-REP1 ± WPRE in C57BL/6J mice.

(A–D) Confocal stacks were prepared from histological sections from eyes injected with AAV2-REP1 at 1E+08 gc (A and B) and 1E+09 gc (C and D) processed at 5 weeks post-injection. Human REP1 immunostaining (purple) and nuclear labeling with Hoechst (blue) were overlaid with the differential interference contrast (DIC) image to demonstrate the retinal layers. Scale bar, 50 µm. (E–H) Representative confocal slices were prepared from histological sections of an AAV2-REP1-WPRE injected eye (1E+09 gc). Human REP1 immunostaining (G) was overlaid with nuclear labeling (F) and DIC (E) to show expression of human REP1 up to the inner layers of retina. Scale bar, 25 µm. Detached areas are an artifact of the processing steps. GCL, ganglion cell layer; IPL, inner plexiform layer; INL, inner nuclear layer; ONL, outer nuclear layer; IS+OS, inner and outer segments of photoreceptors; RPE, retinal pigment epithelium.

Histological analysis of REP1-injected mice eyes confirmed the RPE to be the main target of AAV2 tropism following subretinal delivery of both vectors tested (Figure 6). No human REP1 was detected in the lower-dose cohort following immunostaining of histological sections (data not shown) despite successful WB detection of REP1 in other injected eyes of this cohort. In the higher-dose cohort, however, the WPRE effect was readily apparent when sections were analyzed (Figures 6A–6D). Sample unmasking revealed that the presence of the WPRE enabled human REP1 expression in all retinal layers (Figures 6F–6H). As seen previously with GFP, human REP1 expression was extensive in photoreceptor cells in the ONL and in the RPE. These results show for the first time that subretinal delivery of an AAV2 vector coding the *CHM* gene with the WPRE leads to transduction of all retina layers up to the ganglion cells.

DISCUSSION

The efficacy of an AAV retinal gene therapy approach is mainly dependent on three factors: the expression cassette, the delivery route, and the dose administered.[34] Once the delivery route is optimized, efficacy of the treatment will rely on the number of vector particles that successfully transduce the target cells and how effective the transgenes are in encouraging the cells to express their message. An AAV transgene expression cassette must include a powerful promoter for the gene of interest and a polyadenylation signal, flanked by identical terminal repeats. For its expression to be maximized, a PRE may be included. The WPRE has been shown to enhance GFP expression following AAV transduction in both transformed (293 and HSF8) and primary cells (fibroblasts) in vitro when compared to a non-WPRE construct.[19] Its inclusion in the vector for subthalamic glutamic acid decarboxylase (GAD) gene therapy in a Parkinson's disease rat model[35] paved the way for approval by the US Food and Drug Administration (FDA) to be part of a human gene therapy clinical trial for Parkinson's disease, which proved to be safe some years later.[36] This same sequence was included in the first gene therapy trial for choroideremia in 2012 after the vector had been shown not to be toxic when injected in wild-type and $Chm^{null/WT}$ retinae at doses of 1E+09 gc and 2E+09 gc, respectively.[28] However, until now, the effect of the WPRE in retinal cell transduction has not been elucidated.

We carried out in vitro AAV vector validation experiments that showed for the first time that the WPRE effect is not limited to GFP; both GFP and REP1 expression levels improved in the presence of the WPRE, and this effect was sustained over time. Despite the use of identical backbone constructs, GFP protein levels were consistently higher than REP1 levels. This may be due to the fact that GFP is a smaller, completely exogenous mammalian cell protein, while REP1 is expressed ubiquitously and may undergo greater cellular processing in addition to being much larger (653 amino acids). Following subretinal delivery in mice, GFP-containing vectors showed fluorescence as early as 1 week post-injection for both doses tested. Histological analysis has shown that both transgenes were extensively expressed in the RPE and the ONL when injected at a dose of 1E+09 gc. Although this finding has been reported before using GFP,[28] we now report massive expression of REP1 in the photoreceptor layer following subretinal injection of AAV2-REP1-WPRE, as well as REP1 expression in the inner layers of the retina. An AAV2 vector carrying the human cDNA for REP1 has previously been shown to transduce RPE and photoreceptor cells in WT mice, but at a much higher dose of 2.7E+10 gc.[37] Additionally, human REP1 staining has been reported in the RPE and

photoreceptor inner segments of *Chm*$^{null/WT}$ following delivery of 1E+09 gc of a transgene containing no WPRE and packaged in a different serotype: AAV8.hCHM.[38] Our vector utilizes a serotype 2 capsid and the WPRE sequence, which explains the higher levels of expression achieved at a 10-fold-lower dose.

In our low-dose GFP-injected cohort, the presence of the WPRE clearly makes a difference to expression of GFP; an equivalent level of GFP expression was achieved using the +WPRE vector at a lower dose (2E+08 gc) as it was for the −WPRE vector at a higher dose (1E+09 gc). For the low-dose REP1-injected cohort, no exogenous human REP1 protein was detected by histological analysis. This is likely because the dose used was lower (1E+08 gc) than that used for the GFP study and because REP1 detection requires extra processing for protein labeling with fluorophores. GFP and REP1 protein were detected by WB in the separated eyecups and neural retinae of injected mice at both doses, suggesting a lack of sensitivity in the immunostaining method of REP1 detection.

We have previously shown that AAV2 has the capability of transducing human retinal explants cultured ex vivo.[28,39] Here, we report that the WPRE also improves GFP expression following transduction in retinectomized human retinal tissue, in spite of its inherent atrophic nature. This evidence is of value, as in many cases of inherited retinal disease, retinal tissue may be in a similarly advanced state of degeneration at the time of treatment.

Altogether, our data show that the inclusion of a WPRE sequence within an AAV2 expression cassette significantly enhances expression of target protein in the retina. The effects are most marked at lower doses, where patchy transduction of the retinal layers becomes contiguous. This enhanced effect may be particularly useful in cases where surgical delivery is incomplete due to vector reflux or where a patient's immunological status may lead to less efficient retinal transduction following subretinal AAV administration. Ultimately, inclusion of the WPRE in the transgene expression cassette supports the use of an overall lower dose of vector for retinal gene therapy.

MATERIALS AND METHODS

Plasmid Construction and Vector Production

A plasmid vector containing *CHM* cDNA under the control of a CAG promoter [pAAV2-REP1-WPRE][31] was digested with *ClaI* for excision of the WPRE sequence and religated. Humanized GFP coding sequence was amplified from pTR-UF6[40,41] and cloned into both plasmid vectors by replacing the *CHM* coding sequence at PstI/HindIII cloning sites. All four constructs (pAAV2-REP1, pAAV2-REP1-WPRE, pAAV2-GFP, and pAAV2-GFP-WPRE) were packaged into recombinant AAV serotype 2 by transient polyethylenimine (PEI, Sigma-Aldrich) cotransfection of 293T cells seeded in HYPERFlasks (Sigma-Aldrich).[32] Viral vectors were purified from the cell lysates using iodixanol gradient centrifugation and then concentrated by buffer exchange, resuspended in sterile PBS, and titrated by qPCR using primers designed to amplify a 72-bp fragment within the poly(A) region using supercoiled vector plasmid as standard.

Cell Culture and In Vitro Transduction

293T cells (#12022001, Culture Collections, Public Health England) were cultured in DMEM supplemented with L-glutamine (2 mM), penicillin (100 U/mL), streptomycin (100 μg/mL), and 10% fetal bovine serum. 293 cells (#85120602, Culture Collections, Public Health England) were cultured in minimum essential medium (MEM) supplemented with L-glutamine (2 mM), penicillin (100 U/mL), streptomycin (100 μg/mL), non-essential amino acids (1%), and 10% fetal bovine serum. Cells were maintained at 37°C in a 5% CO_2 environment. For in vitro transduction experiments, cells were seeded in 12-well plates at a density of 3E+05 cells per well on the day prior to transduction. Transduction with AAV was performed at a range of MOIs, and cells were harvested at 1 and 5 days post-transduction in RIPA buffer (Millipore) supplemented with protease inhibitors (cOmplete Mini, Roche) for protein analysis.

Mice

All animal experiments were performed in compliance with local and national ethical and legal authorities and in accordance with the Association for Research in Vision and Ophthalmology statements on the care and use of animals in ophthalmic research. JAX C57BL/6J mice were purchased from Charles River Laboratories and housed in the Biomedical Sciences Division (University of Oxford, Oxford, UK). Animals were kept in a 12-hr light/12-hr dark cycle, with food and water available ad libitum. All experiments were conducted in 6- to 8-week-old female mice, and surgery and in vivo imaging were performed under general anesthesia. Animals were anesthetized by intraperitoneal injection of ketamine (Vetalar, 80 mg/kg body weight) and xylazine (Rompun, 10 mg/kg body weight) and the pupils fully dilated with tropicamide 1% and phenylephrine hydrochloride 2.5% eye drops (both from Bausch & Lomb). Proxymetacaine hydrochloride 0.5% eye drops were used for additional topical anesthesia. Anaesthesia was reversed following procedures by intraperitoneal injection of atipamezole (Antisedan, 2 mg/kg body weight).

Subretinal Injections

Mice pupils were dilated as above and a circular 6-mm coverglass was applied onto the cornea with a gel lubricant (Viscotears, Novartis) to allow visualization of the fundus. Injections were performed with a 35G needle mounted on a NanoFil 10-μL syringe (WPI) under direct visual control using a surgical microscope (M620 F20, Leica). The superior rectus muscle was hold with notched forceps to keep eye position stable. 2 μL vector suspension was delivered into the subretinal space. One eye of each animal received a WPRE-containing vector, and its corresponding non-WPRE version was delivered into the contralateral eye. Different syringes and needles were used for different viral preparations, and the needle was flushed with PBS between individual injections.

Fundus Imaging Using a cSLO

Standardized mouse AF imaging using a cSLO (Spectralis HRA, Heidelberg Engineering) was performed in all animals according to a previously published protocol.[29,30] All images were recorded using the 55° lens of the Spectralis HRA. Images were recorded using the

"automatic real time" (ART) mode and a standardized signal detector sensitivity. For quantitative analysis of fundus AF, a two-pixel radius (σ) Gaussian blur was applied to fundus AF images and the mean gray level measured within a ring-shaped area located at radii between 650 and 900 pixels from the optic disc center using ImageJ software (NIH).

Tissue Collection and Processing

Mouse eyes for histology were dissected in PBS. After enucleation, the cornea and lens were removed under direct visualization with a surgical microscope. Eyecups were fixed in 4% paraformaldehyde for 30 min and then cryoprotected using a 10%–30% sucrose gradient. Samples were embedded in optimal cutting temperature (OCT) compound (VWR), frozen on dry ice and stored at −80°C until sectioning. Sections were cut at 18 μm thickness, air-dried, counterstained with Hoechst 33342 (1:4,000), covered with ProLong Gold antifade mounting media (both from Thermo-Fisher Scientific) and examined under a confocal microscope.

Western Blot Analysis

Samples from 293-transduced in vitro experiments were lysed in radioimmunoprecipitation (RIPA) buffer supplemented with protease inhibitors, and sonicated for protein extraction. Mouse neural retinae and remaining eyecups were collected separately in RIPA buffer supplemented with protease inhibitors and frozen at −80°C. Protein was extracted by pestle tissue disruption and the lysate was cleared by centrifugation (17,000 × *g*, 20 min). Total protein in all samples was quantified using the bicinchoninic assay (Thermo-Fisher Scientific), and loaded in a gel (see Tables S1 and S2). Samples were subjected to SDS-PAGE on a 7.5% or 10% pre-cast polyacrylamide gel (Criterion, Bio-Rad) and transferred to a PVDF membrane (TransBlot Turbo, Bio-Rad). Membranes were blocked with PBS + 0.1% Tween 20 (PBST) + 3% BSA for 45 min and incubated with monoclonal antibodies for 1 hr (see Tables S1 and S2). Membranes were washed three times for 7 min each with PBST, incubated with secondary antibodies, washed again as before, and detected using Clarity ECL (Bio-Rad) and an Odyssey Imaging System (LI-COR Biosciences). Densitometry data analysis was performed using the ImageStudio Lite software (LI-COR Biosciences).

Histology and Immunohistochemistry

Retinal sections were hydrated in PBS 3x5 min, and blocked for 1 hr at room temperature in PBS+0.1% Triton X-100+10% donkey serum. Sections were incubated overnight at 4°C with the primary antibody, and then for 1 hr at room temperature with the species-appropriate secondary antibody. Antibody dilutions were prepared in PBS+0.1% Triton X-100+1% donkey serum (see Table S3). After each step, sections were rinsed three times for 5 min each with PBS+0.05% Tween 20. All sections were counterstained with Hoechst 33342 and mounted with ProLong Gold.

Human Retinal Explant Culture

Retinal tissue was obtained with patient consent and Research Ethics Committee Approval (REC reference no. 10/H0505) from two patients undergoing clinically indicated retinectomy. In each case, 23G three-port pars plana vitrectomy was performed and the tissue removed either as individual discs using the vitrector at 100 cuts per minute (Ocutome, Alcon Surgical) with manual aspiration or en bloc using vitreoretinal forceps. In the latter case, the tissue was subsequently divided under sterile conditions with a scalpel blade. Within 1 hr of tissue collection, retinal fragments were transferred using a 3-mL Pasteur pipette into individual organotypic culture inserts (BD Falcon), which were in turn placed within a 24-well plate. Samples were cultured in 700 μL of a Neurobasal-A based culture medium supplemented with L-glutamine (0.8 mM), penicillin (100 U/mL), streptomycin (100 μg/mL), B27 supplement (2%), and N2 supplement (1%) (all from Thermo-Fisher Scientific), and incubated at 34°C in a 5% CO_2 environment. After 24 hr, 300 μL of media within each insert was exchanged for fresh media and, in a blinded fashion, 10E+10 gc of either AAV2-GFP or AAV2-GFP-WPRE was added to separate wells. An additional well for each patient was allocated as an untransduced control. Subsequently, 300 μL of media contained within each insert was replaced with fresh media every 48 hr for 12 days.

Confocal Microscopy

Murine retinal sections were viewed on a Zeiss LSM-710 inverted confocal microscope (Zeiss). The injected area was located using epifluorescence illumination before taking a series of XY optical sections. Fluorescence images were acquired using 350-nm UV, 488-nm argon, and the 543-nm HeNe excitation lasers, as appropriate. Images were processed using ImageJ software.

Fluorescence Imaging of Human Retinal Explants

Explants were imaged on alternate days up to 12 days following viral transduction with an inverted epifluorescence microscope (DMIL, Leica) under standardized conditions of ambient lighting, gain, and exposure. Fluorescence was quantified using ImageJ software through calculation of the mean gray level of tissue within 8-bit images. For this, the largest region of unfolded retina was defined on the transmission image and this region of interest applied to the corresponding fluorescence micrograph. A two-pixel σ Gaussian blur was then applied and the mean gray level for the region calculated. To correct for background fluorescence, normalized gray level was calculated by subtraction of the mean gray level measured from the untransduced explant from the same patient.

Statistical Analysis

Transgene expression in vitro was compared using a two-way ANOVA with MOI and WPRE as factors. Mean gray levels on cSLO images were compared using two-way repeated-measures ANOVA with time and WPRE as factors. Transgene expression in mouse eyecups and retinas were compared using a two-way ANOVA with injected AAV and dose as factors. The Bonferroni test was applied in all instances to correct for multiple testing, with a 95% confidence interval. Mean ± SEM values are shown in all figures. All statistical analysis was done using Prism 7 for Windows.

SUPPLEMENTAL INFORMATION

Supplemental Information includes three tables and can be found with this article online at http://dx.doi.org/10.1016/j.omtn.2016.12.006.

AUTHOR CONTRIBUTIONS

Conceptualization: M.I.P., A.R.B., and R.E.M.; Methodology: M.I.P., A.R.B., H.O.O., M.E.M., and R.E.M.; Formal Analysis: M.I.P., A.R.B., H.O.O., and R.E.M.; Investigation: M.I.P., A.R.B., H.O.O., and R.E.M.; Writing – Original Draft: M.I.P.; Writing – Review & Editing: M.I.P., A.R.B., H.O.O., M.E.M., and R.E.M.; Supervision: A.R.B. and R.E.M.; Funding Acquisition: R.E.M.

CONFLICTS OF INTEREST

R.E.M. is a director of NightstaRx, a choroideremia gene therapy company established by the University of Oxford and based at the Wellcome Trust Building (215 Euston Road, London NW1 2BE, UK). The remaining authors declare no competing financial interests.

ACKNOWLEDGMENTS

This research was funded by Fight for Sight, the Wellcome Trust, the Health Foundation, the Medical Research Council, the Royal College of Surgeons of Edinburgh, and the NIHR Ophthalmology and Oxford Biomedical Research Centre.

REFERENCES

1. Lipinski, D.M., Thake, M., and MacLaren, R.E. (2013). Clinical applications of retinal gene therapy. Prog. Retin. Eye Res. *32*, 22–47.
2. Wu, Z., Yang, H., and Colosi, P. (2010). Effect of genome size on AAV vector packaging. Mol. Ther. *18*, 80–86.
3. Willett, K., and Bennett, J. (2013). Immunology of AAV-mediated gene transfer in the eye. Front. Immunol. *4*, 261.
4. Ali, R.R., Reichel, M.B., Thrasher, A.J., Levinsky, R.J., Kinnon, C., Kanuga, N., Hunt, D.M., and Bhattacharya, S.S. (1996). Gene transfer into the mouse retina mediated by an adeno-associated viral vector. Hum. Mol. Genet. *5*, 591–594.
5. Auricchio, A., Kobinger, G., Anand, V., Hildinger, M., O'Connor, E., Maguire, A.M., Wilson, J.M., and Bennett, J. (2001). Exchange of surface proteins impacts on viral vector cellular specificity and transduction characteristics: the retina as a model. Hum. Mol. Genet. *10*, 3075–3081.
6. Yang, G.S., Schmidt, M., Yan, Z., Lindbloom, J.D., Harding, T.C., Donahue, B.A., Engelhardt, J.F., Kotin, R., and Davidson, B.L. (2002). Virus-mediated transduction of murine retina with adeno-associated virus: effects of viral capsid and genome size. J. Virol. *76*, 7651–7660.
7. Charbel Issa, P., De Silva, S.R., Lipinski, D.M., Singh, M.S., Mouravlev, A., You, Q., Barnard, A.R., Hankins, M.W., During, M.J., and Maclaren, R.E. (2013). Assessment of tropism and effectiveness of new primate-derived hybrid recombinant AAV serotypes in the mouse and primate retina. PLoS ONE *8*, e60361.
8. Boye, S.E., Boye, S.L., Lewin, A.S., and Hauswirth, W.W. (2013). A comprehensive review of retinal gene therapy. Mol. Ther. *21*, 509–519.
9. Jacobson, S.G., Acland, G.M., Aguirre, G.D., Aleman, T.S., Schwartz, S.B., Cideciyan, A.V., Zeiss, C.J., Komaromy, A.M., Kaushal, S., Roman, A.J., et al. (2006). Safety of recombinant adeno-associated virus type 2-RPE65 vector delivered by ocular subretinal injection. Mol. Ther. *13*, 1074–1084.
10. Bainbridge, J.W., Smith, A.J., Barker, S.S., Robbie, S., Henderson, R., Balaggan, K., Viswanathan, A., Holder, G.E., Stockman, A., Tyler, N., et al. (2008). Effect of gene therapy on visual function in Leber's congenital amaurosis. N. Engl. J. Med. *358*, 2231–2239.
11. Maguire, A.M., Simonelli, F., Pierce, E.A., Pugh, E.N., Jr., Mingozzi, F., Bennicelli, J., Banfi, S., Marshall, K.A., Testa, F., Surace, E.M., et al. (2008). Safety and efficacy of gene transfer for Leber's congenital amaurosis. N. Engl. J. Med. *358*, 2240–2248.
12. Hauswirth, W.W., Aleman, T.S., Kaushal, S., Cideciyan, A.V., Schwartz, S.B., Wang, L., Conlon, T.J., Boye, S.L., Flotte, T.R., Byrne, B.J., and Jacobson, S.G. (2008). Treatment of leber congenital amaurosis due to RPE65 mutations by ocular subretinal injection of adeno-associated virus gene vector: short-term results of a phase I trial. Hum. Gene Ther. *19*, 979–990.
13. MacLaren, R.E., Groppe, M., Barnard, A.R., Cottriall, C.L., Tolmachova, T., Seymour, L., Clark, K.R., During, M.J., Cremers, F.P., Black, G.C., et al. (2014). Retinal gene therapy in patients with choroideremia: initial findings from a phase 1/2 clinical trial. Lancet *383*, 1129–1137.
14. Edwards, T.L., Jolly, J.K., Groppe, M., Barnard, A.R., Cottriall, C.L., Tolmachova, T., Black, G.C., Webster, A.R., Lotery, A.J., Holder, G.E., et al. (2016). Visual acuity after retinal gene therapy for choroideremia. N. Engl. J. Med. *374*, 1996–1998.
15. Ghazi, N.G., Abboud, E.B., Nowilaty, S.R., Alkuraya, H., Alhommadi, A., Cai, H., Hou, R., Deng, W.T., Boye, S.L., Almaghamsi, A., et al. (2016). Treatment of retinitis pigmentosa due to MERTK mutations by ocular subretinal injection of adeno-associated virus gene vector: results of a phase I trial. Hum. Genet. *135*, 327–343.
16. Rakoczy, E.P., Lai, C.-M., Magno, A.L., Wikstrom, M.E., French, M.A., Pierce, C.M., Schwartz, S.D., Blumenkranz, M.S., Chalberg, T.W., Degli-Esposti, M.A., and Constable, I.J. (2015). Gene therapy with recombinant adeno-associated vectors for neovascular age-related macular degeneration: 1 year follow-up of a phase 1 randomised clinical trial. Lancet *386*, 2395–2403.
17. Bainbridge, J.W.B., Mehat, M.S., Sundaram, V., Robbie, S.J., Barker, S.E., Ripamonti, C., Georgiadis, A., Mowat, F.M., Beattie, S.G., Gardner, P.J., et al. (2015). Long-term effect of gene therapy on Leber's congenital amaurosis. N. Engl. J. Med. *372*, 1887–1897.
18. Georgiadis, A., Duran, Y., Ribeiro, J., Abelleira-Hervas, L., Robbie, S.J., Sünkel-Laing, B., Fourali, S., Gonzalez-Cordero, A., Cristante, E., Michaelides, M., et al. (2016). Development of an optimized AAV2/5 gene therapy vector for Leber congenital amaurosis owing to defects in RPE65. Gene Ther. *23*, 857–862.
19. Loeb, J.E., Cordier, W.S., Harris, M.E., Weitzman, M.D., and Hope, T.J. (1999). Enhanced expression of transgenes from adeno-associated virus vectors with the woodchuck hepatitis virus posttranscriptional regulatory element: implications for gene therapy. Hum. Gene Ther. *10*, 2295–2305.
20. Zufferey, R., Donello, J.E., Trono, D., and Hope, T.J. (1999). Woodchuck hepatitis virus posttranscriptional regulatory element enhances expression of transgenes delivered by retroviral vectors. J. Virol. *73*, 2886–2892.
21. Paterna, J.-C., Moccetti, T., Mura, A., Feldon, J., and Büeler, H. (2000). Influence of promoter and WHV post-transcriptional regulatory element on AAV-mediated transgene expression in the rat brain. Gene Ther. *7*, 1304–1311.
22. Xu, R., Janson, C.G., Mastakov, M., Lawlor, P., Young, D., Mouravlev, A., Fitzsimons, H., Choi, K.L., Ma, H., Dragunow, M., et al. (2001). Quantitative comparison of expression with adeno-associated virus (AAV-2) brain-specific gene cassettes. Gene Ther. *8*, 1323–1332.
23. Klein, R.L., Hamby, M.E., Gong, Y., Hirko, A.C., Wang, S., Hughes, J.A., King, M.A., and Meyer, E.M. (2002). Dose and promoter effects of adeno-associated viral vector for green fluorescent protein expression in the rat brain. Exp. Neurol. *176*, 66–74.
24. Johansen, J., Tornøe, J., Møller, A., and Johansen, T.E. (2003). Increased in vitro and in vivo transgene expression levels mediated through cis-acting elements. J. Gene Med. *5*, 1080–1089.
25. Ylä-Herttuala, S. (2012). Endgame: glybera finally recommended for approval as the first gene therapy drug in the European union. Mol. Ther. *20*, 1831–1832.
26. Schambach, A., Galla, M., Maetzig, T., Loew, R., and Baum, C. (2007). Improving transcriptional termination of self-inactivating gamma-retroviral and lentiviral vectors. Mol. Ther. *15*, 1167–1173.
27. Higashimoto, T., Urbinati, F., Perumbeti, A., Jiang, G., Zarzuela, A., Chang, L.-J., Kohn, D.B., and Malik, P. (2007). The woodchuck hepatitis virus post-transcriptional regulatory element reduces readthrough transcription from retroviral vectors. Gene Ther. *14*, 1298–1304.
28. Tolmachova, T., Tolmachov, O.E., Barnard, A.R., de Silva, S.R., Lipinski, D.M., Walker, N.J., Maclaren, R.E., and Seabra, M.C. (2013). Functional expression of

Rab escort protein 1 following AAV2-mediated gene delivery in the retina of choroideremia mice and human cells ex vivo. J. Mol. Med. (Berl.) *91*, 825–837.

29. Charbel Issa, P., Singh, M.S., Lipinski, D.M., Chong, N.V., Delori, F.C., Barnard, A.R., and MacLaren, R.E. (2012). Optimization of in vivo confocal autofluorescence imaging of the ocular fundus in mice and its application to models of human retinal degeneration. Invest. Ophthalmol. Vis. Sci. *53*, 1066–1075.

30. Charbel Issa, P., Barnard, A.R., Singh, M.S., Carter, E., Jiang, Z., Radu, R.A., Schraermeyer, U., and MacLaren, R.E. (2013). Fundus autofluorescence in the Abca4(-/-) mouse model of Stargardt disease–correlation with accumulation of A2E, retinal function, and histology. Invest. Ophthalmol. Vis. Sci. *54*, 5602–5612.

31. Barnard, A.R., Groppe, M., and MacLaren, R.E. (2014). Gene therapy for choroideremia using an adeno-associated viral (AAV) vector. Cold Spring Harb. Perspect. Med. *5*, a017293.

32. Lawlor, P.A., Bland, R.J., Mouravlev, A., Young, D., and During, M.J. (2009). Efficient gene delivery and selective transduction of glial cells in the mammalian brain by AAV serotypes isolated from nonhuman primates. Mol. Ther. *17*, 1692–1702.

33. Johnson, T.V., and Martin, K.R. (2008). Development and characterization of an adult retinal explant organotypic tissue culture system as an in vitro intraocular stem cell transplantation model. Invest. Ophthalmol. Vis. Sci. *49*, 3503–3512.

34. MacLaren, R.E. (2015). Gene therapy for retinal disease: what lies ahead. Ophthalmologica *234*, 1–5.

35. Luo, J., Kaplitt, M.G., Fitzsimons, H.L., Zuzga, D.S., Liu, Y., Oshinsky, M.L., and During, M.J. (2002). Subthalamic GAD gene therapy in a Parkinson's disease rat model. Science *298*, 425–429.

36. Kaplitt, M.G., Feigin, A., Tang, C., Fitzsimons, H.L., Mattis, P., Lawlor, P.A., Bland, R.J., Young, D., Strybing, K., Eidelberg, D., and During, M.J. (2007). Safety and tolerability of gene therapy with an adeno-associated virus (AAV) borne GAD gene for Parkinson's disease: an open label, phase I trial. Lancet *369*, 2097–2105.

37. Vasireddy, V., Mills, J.A., Gaddameedi, R., Basner-Tschakarjan, E., Kohnke, M., Black, A.D., Alexandrov, K., Zhou, S., Maguire, A.M., Chung, D.C., et al. (2013). AAV-mediated gene therapy for choroideremia: preclinical studies in personalized models. PLoS ONE *8*, e61396.

38. Black, A., Vasireddy, V., Chung, D.C., Maguire, A.M., Gaddameedi, R., Tolmachova, T., Seabra, M., and Bennett, J. (2014). Adeno-associated virus 8-mediated gene therapy for choroideremia: preclinical studies in in vitro and in vivo models. J. Gene Med. *16*, 122–130.

39. De Silva, S.R., Charbel Issa, P., Singh, M.S., Lipinski, D.M., Barnea-Cramer, A.O., Walker, N.J., Barnard, A.R., Hankins, M.W., and MacLaren, R.E. (2016). Single residue AAV capsid mutation improves transduction of photoreceptors in the Abca4(-/-) mouse and bipolar cells in the rd1 mouse and human retina ex vivo. Gene Ther. *23*, 767–774.

40. Zolotukhin, S., Potter, M., Hauswirth, W.W., Guy, J., and Muzyczka, N. (1996). A "humanized" green fluorescent protein cDNA adapted for high-level expression in mammalian cells. J. Virol. *70*, 4646–4654.

41. Zolotukhin, S., Byrne, B.J., Mason, E., Zolotukhin, I., Potter, M., Chesnut, K., Summerford, C., Samulski, R.J., and Muzyczka, N. (1999). Recombinant adeno-associated virus purification using novel methods improves infectious titer and yield. Gene Ther. *6*, 973–985.

OMTN, Volume *6*

Supplemental Information

Inclusion of the Woodchuck Hepatitis Virus Posttranscriptional Regulatory Element Enhances AAV2-Driven Transduction of Mouse and Human Retina

Maria I. Patrício, Alun R. Barnard, Harry O. Orlans, Michelle E. McClements, and Robert E. MacLaren

Supplemental Methods

Table S1 – Total protein loading amounts and antibodies dilutions' used for Western blot analysis for AAV2-GFP±WPRE treated samples.

**Antibody/Sample type AAV2-GFP±WPRE**	AAV-transduced 293 cells	AAV-injected eye cups	AAV-injected retinas
Total amount of protein loaded	5 µg	1 µg	4 µg
Dilution of anti-GFP (ab32146, Abcam, Cambridge, UK)	1:20,000	1:10,000	1:10,000
Dilution of anti-β-Actin (AM4302, Thermo-Fisher Scientific, Loughborough, UK)	1:20,000	1:10,000	1:10,000
Dilution of Donkey anti-mouse HRP (ab98799, Abcam, Cambridge, UK)	1:10,000	1:10,000	1:10,000
Dilution of Donkey anti-rabbit HRP (ab98503, Abcam, Cambridge, UK)	1:10,000	1:10,000	1:10,000

Table S2 - Total protein loading amounts and antibodies dilutions' used for Western blot analysis for AAV2-REP1±WPRE treated samples.

**Antibody/Sample type AAV2-REP1±WPRE**	AAV-transduced 293 cells	AAV-injected eye cups	AAV-injected retinas
Total amount of protein loaded	10 µg	10 µg	25 µg
Dilution of anti-human REP1 (MABN52, Millipore, Watford, UK)	1:2,500	1:1,000	1:1,000
Dilution of anti-β-Actin (AM4302, Thermo-Fisher Scientific, Loughborough, UK)	1:50,000	1:50,000	1:50,000
Dilution of Donkey anti-mouse HRP (ab98799, Abcam, Cambridge, UK)	1:10,000	1:10,000	1:10,000
Dilution of Donkey anti-rabbit HRP (ab98503, Abcam, Cambridge, UK)	1:10,000	1:10,000	1:10,000

Table S3 – List of antibodies used in immunohistochemistry analysis.

**Antibodies**	Dilution
Anti-cone arrestin (AB15282, Millipore, Watford, UK)	1:500
Anti-GFAP (ab7779, Abcam, Cambridge, UK)	1:500
Anti-CHM (HPA003231, Sigma, Gillingham, UK)	1:1,000
Donkey anti-rabbit Alexa Fluor 568 (A10042, Thermo-Fisher Scientific, Loughborough, UK)	1:500
